# *Salmonella spvC* Gene Inhibits Pyroptosis and Intestinal Inflammation to Aggravate Systemic Infection in Mice

**DOI:** 10.3389/fmicb.2020.562491

**Published:** 2020-12-15

**Authors:** Lingli Zuo, Liting Zhou, Chaoyi Wu, Yanlin Wang, Yuanyuan Li, Rui Huang, Shuyan Wu

**Affiliations:** ^1^Department of Medical Microbiology, School of Biology and Basic Medical Science, Medical College of Soochow University, Suzhou, China; ^2^Medical Research Center, The People's Hospital of Suzhou New District, Suzhou, China

**Keywords:** *Salmonella*, *spvC*, pyroptosis, intestinal inflammation, bacterial dissemination

## Abstract

*Salmonella enterica* serovar Typhimurium (*S*). Typhimurium is a primary foodborne pathogen infecting both humans and animals. *Salmonella* plasmid virulence C (*spvC*) gene is closely related to *S*. Typhimurium dissemination in mice, while the mechanisms remain to be fully elucidated. Pyroptosis, a gasdermin-mediated inflammatory cell death, plays a role in host defense against bacterial infection, whereas the effect of *spvC* on pyroptosis and its function in inflammatory injury induced by *S*. Typhimurium are rather limited. In our study, C57BL/6 mice and J774A.1 cells infected with *S*. Typhimurium wild-type strain SL1344, *spvC* deletion mutant, *spvC* K136A site-directed mutant, and complemented strain were used to investigate potential pathogenesis of *spvC*. We verity that SpvC attenuates intestinal inflammation, suppresses pyroptosis through phosphothreonine lyase activity, and reduces pyroptosis in the ceca. Moreover, the reduction of inflammation via *spvC* results in systemic infection. These findings demonstrate that *spvC* inhibits pyroptosis and intestinal inflammation to promote bacterial dissemination, which provide new strategies for controlling systemic infection caused by *Salmonella* and novel insights for the treatment of other corresponding diseases.

## Introduction

*Salmonella*, a facultative intracellular Gram-negative bacterium, is responsible for a wide range of food- and water-borne diseases ranging from gastroenteritis to typhoid fever depending on hosts and serotypes. *Salmonella* is responsible for a recent global estimated 9% cases of diarrhea annually, which results in disproportionate 41% of all diarrheal-associated mortality (Besser, 2018). The pathogenic factors of *Salmonella* include endotoxin, enterotoxin, and virulent effectors encoded by bacterial genetic elements. Most pathogenic *Salmonella* harbor a pSLT virulence plasmid of around 90 kb that contains a highly conserved 8-kb region of five genes (*spvRABCD*), which have been reported to be implicated in intracellular survival and growth (Guiney and Fierer, 2011; Passaris et al., 2018). Previously, we showed that *Salmonella spvB* gene could reduce host cell autophagy and regulate intracellular iron homeostasis (Chu et al., 2016; Yang et al., 2019). *spvC* is another essential factor of *Salmonella* virulence determinant, and its encoding product, SpvC, shares 63% identity at the amino acid level with OspF of *Shigella flexneri* and exhibits the same phosphothreonine lyase activity on host mitogen-activated protein kinase (MAPK). *spvC* leads to attenuation of the intestinal inflammatory response, which is thought to be important during systemic infection of *Salmonella* (Li et al., 2007; Mazurkiewicz et al., 2008; Haneda et al., 2012).

Pyroptosis, a programmed cell death (PCD) mediated by gasdermin family proteins, is characterized by cell swelling; and the terminal event is represented by rupture of the cell membrane, causing release of cytoplasmic contents of the cell, including pro-inflammatory cytokines, endogenous ligands, alarmins, and other danger-associated molecular patterns (DAMPs) (Gaidt and Hornung, 2016). Pyroptosis is regulated via a Caspase-1-dependent or Caspase-1-independent pathway; the latter is mediated by human Caspase-4, Caspase-5, or mouse Caspase-11 (Man et al., 2017). Canonical inflammasome pathway is activated in response to cytosolic pattern recognition receptors (PRRs) such as nucleotide-binding oligomerization domain, leucine-rich repeat and pyrin domain-containing 3 (NLRP3), and NLR with CARD domain-containing 4 (NLRC4). These receptors assemble Procaspase-1 into multiprotein complexes, which serve as Caspase-1-activating platforms. On the other hand, the non-canonical inflammasome pathway results in the cleavage of Caspase-4/5/11. Caspase-1/4/5/11 functioned as “molecular scissors” cleave not only gasdermin D (GSDMD) but also interleukin (IL)-1β (IL-1β) and IL-18 (Feng et al., 2018; Rathinam et al., 2019). Gasdermin family comprises six paralogous genes: *GSDMA, GSDMB, GSDMC, GSDMD, GSDME* (also known as *DFNA5*), and *PJVK* (also known as *DFNB59*) and are components of a cell death program (Broz et al., 2020) Both gasdermin B (GSDMB) and gasdermin E (GSMDE) act as tumor suppressors via triggering pyroptosis and promote tumor clearance, while GSDMD is a crucial executor of pyroptosis in anti-infection immunity (Pandeya et al., 2019; Wang et al., 2020; Zhou et al., 2020). Binding of the GSDMD N terminal (GSDMD-NT) domain β1–β2 loops to a common hydrophobic pocket in the GSDMD C terminal (GSDMD-CT) domain compromises auto-inhibition (Liu et al., 2019). The activated fragment GSDMD-NT oligomerizes in the cell membrane by specific binding to phosphoinositides and cardiolipin to form a pore of 10–16 nm in diameter, through which substrates of a smaller diameter, such as IL-1β, are secreted. Host cells not only expose the intracellular pathogens by pyroptosis but also capture the pathogens by pore-induced intracellular traps (PITs), and then neutrophils phagocytize and remove PITs by efferocytosis to prevent the dissemination of bacteria (Jorgensen et al., 2016b; Kovacs and Miao, 2017).

*Salmonella enterica* serovar Typhimurium (*S*. Typhimurium) is one of the most common isolates in *Salmonella*. It could traverse the epithelial cells layer of small intestine, replicate in phagocytes, and disseminate, resulting in systemic infection. In this study, C57BL/6 mice and J774A.1 cells infected with *S*. Typhimurium-carrying, *S*. Typhimurium-free, or site-directed mutant *spvC* were used for *in vivo* and *in vitro* assays. We reveal the contribution of *spvC* to pathogenesis of *S*. Typhimurium via inhibition of pyroptosis and intestinal inflammation to promote bacterial dissemination.

## Materials and Methods

### Bacterial Strains and Growth Conditions

*Salmonella* Typhimurium wild-type strain SL1344 (STM-WT) was kindly provided by Professor Qian Yang (Nanjing Agricultural University, Nanjing, China). STM-WT, *spvC* deletion mutant (STM-Δ*spvC*), and *spvC* site-directed mutant (STM-c-*spvC* K136A), which lacks phosphothreonine lyase activity and complemented strain (STM-c-*spvC*), were grown at 37°C in Luria Bertani (LB, Hangwei, China) broth overnight. STM-c-*spvC* K136A and STM-c-*spvC* were cultured in the media with 100 μg/ml of ampicillin (Sigma, USA). On the day of infection, *S*. Typhimurium were subcultured (1:100) for 3 h at 37°C in fresh LB broth to generate bacteria grown to log phase. Both STM-c-*spvC* K136A and STM-c-*spvC* were supplemented with 0.2% l-arabinose (Sigma, USA). Bacteria were then washed three times in phosphate-buffered saline (PBS), quantified by OD_600_.

### Construction of *spvC* Deletion Mutant, Site-Directed Mutant, and Complemented Strain

The primers used for bacterial strains construction are listed in Supplementary Table 1. STM-Δ*spvC* was constructed using λRed recombination system (Song et al., 2010); and the corresponding plasmids were gifts from Professor Daoguo Zhou (Purdue University, West Lafayette, USA). pBAD/gIII expression system was used to construct STM-c-*spvC* K136A and STM-c-*spvC* (Szeliova et al., 2016). *spvC* K136A allele was amplified by overlap PCR. *spvC* deletion mutant, site-directed mutant, and complemented strain were identified by PCR and sequencing.

### Cell Culture and Bacterial Infection

J774A.1 cells were purchased from the Procell Life Science & Technology Co., Ltd. Cells were routinely cultured in Dulbecco's modified Eagle medium (HyClone Laboratories, Logan, UT, USA) supplemented with 10% (v/v) fetal bovine serum (Biological Industries, Kibbutz Beit-Haemek, Israel) at 37°C in a 5% CO_2_ atmosphere. Cells were seeded in 12-well plates at a density of 1 × 10^6^ cells per well and co-cultured with *S*. Typhimurium at the multiplicity of infection (MOI) of 10:1. Cells were washed with PBS; and fresh medium containing amikacin (100 μg/ml, MilliporeSigma, Burlington, MA, USA) was added to kill the extracellular bacteria for 2 h. Afterwards, infected cells were washed and subsequently cultured in fresh medium containing amikacin (10 μg/ml, MilliporeSigma) to limit extracellular replication of bacteria. Proteins were extracted at 8 hpi using radioimmunoprecipitation assay (RIPA) buffer containing protease inhibitors and phosphatase inhibitors (Beyotime Biotechnology).

### Mice Experiment

Specific-pathogen-free (SPF) C57BL/6 mice aged 6–8 weeks were purchased from the experimental animal center of Soochow University and bred locally. Mice (fasted for 4 h) were given an oral dose of 100 μl, 200 mg/ml of streptomycin (Sigma, USA) and then resumed normal diet. Mice were randomly divided into three groups, including control, STM-WT, and STM-Δ*spvC* infection groups; 1 × 10^8^ colony-forming unit (CFU) *S*. Typhimurium strains, respectively, in 100 μl of PBS were used for oral gavage to mice that were fasted for 4 h before infection. The cecum, liver, spleen, and serum were collected at 24 h post infection (hpi) and 72 hpi for the subsequent experiments.

### Histopathological Analysis

Tissue samples of the liver and cecum from infected mice were washed three times with PBS and fixed in 10% formaldehyde solution at 4°C overnight. Then samples were processed with routine histological procedures, dehydration, paraffin embedding, section cutting, and deparaffinization. The sections were stained with hematoxylin–eosin (Baso, Zhuhai, China) and observed under a light microscope (Olympus, Japan).

### Bacterial Burden Measurement

Fresh liver and spleen were harvested and immersed in 100 μg/ml of amikacin for 1 h, and tissues were homogenized at 0.3% Triton (Sigma, USA) for 30 min. Tissue homogenate was serially diluted 1:10 in soft agar (55°C) and plated on *SS* (*Salmonella*–*Shigella*) agar plates (Hangwei, Hangzhou, China). Colonies were counted after 16 h.

### Western Blot Analysis

Thirty milligrams of the ceca was cut into pieces and lysed in 1 ml of RIPA buffer containing protease inhibitors and phosphatase inhibitors (Beyotime Biotechnology). Samples were homogenized on ice, centrifuged for supernatant at 12,000 rpm for 30 min at 4°C, and heated to 100°C for 5 min. Protein extracts resuspended in sample loading buffer were separated by electrophoresis through 12–15% polyacrylamide gels. Following electrophoretic transfer of proteins onto polyvinylidene difluoride (PVDF) membranes (Millipore), non-specific binding was blocked by incubation with 5% non-fat dry milk (Sangon Biotech Shanghai Co., Ltd.), and then membranes were incubated with primary antibodies anti-phospho-ERK1/2 and anti-phospho-JNK1/2 (1:1,000 dilution, Cell Signaling Technology); anti-NLRP3, anti-NLRC4, anti-Caspase-1, anti-Caspase-11, anti-GSDMD, and anti-histone H3 (1:1,000 dilution, abcam); anti-GAPDH (1:1,000 dilution, Boster); and anti-Tubulin (1:1,000, Beyotime) overnight at 4°C. Membranes were then washed and incubated with the horseradish peroxidase (HRP)-labeled goat anti-rabbit IgG (1:3,000 dilution, Beyotime) for 1 h at room temperature. Proteins were visualized using enhanced chemiluminescence (ECL) reagent (Meilunbio). The grayscale values of the bands were determined by ImageJ launcher broken symmetry software program (National Institutes of Health, Bethesda, MD, USA).

### ELISA

To assess cytokine secretion during infection, C57BL/6 mice were bled, and serum IL-1β levels were assessed by ELISA at 24 and 72 hpi according to the kit instructions. ELISA kits were from Beyotime Biotechnology.

### Statistical Analysis

Statistical significance was determined by independent Student's *t*-test for two groups and ANOVA for three or more groups. *P* < 0.05 was considered to be statistically significant.

## Results

### Effects of *spvC* on *Salmonella* Pathogenesis in Intestinal Inflammatory Response

**Figure 1:**
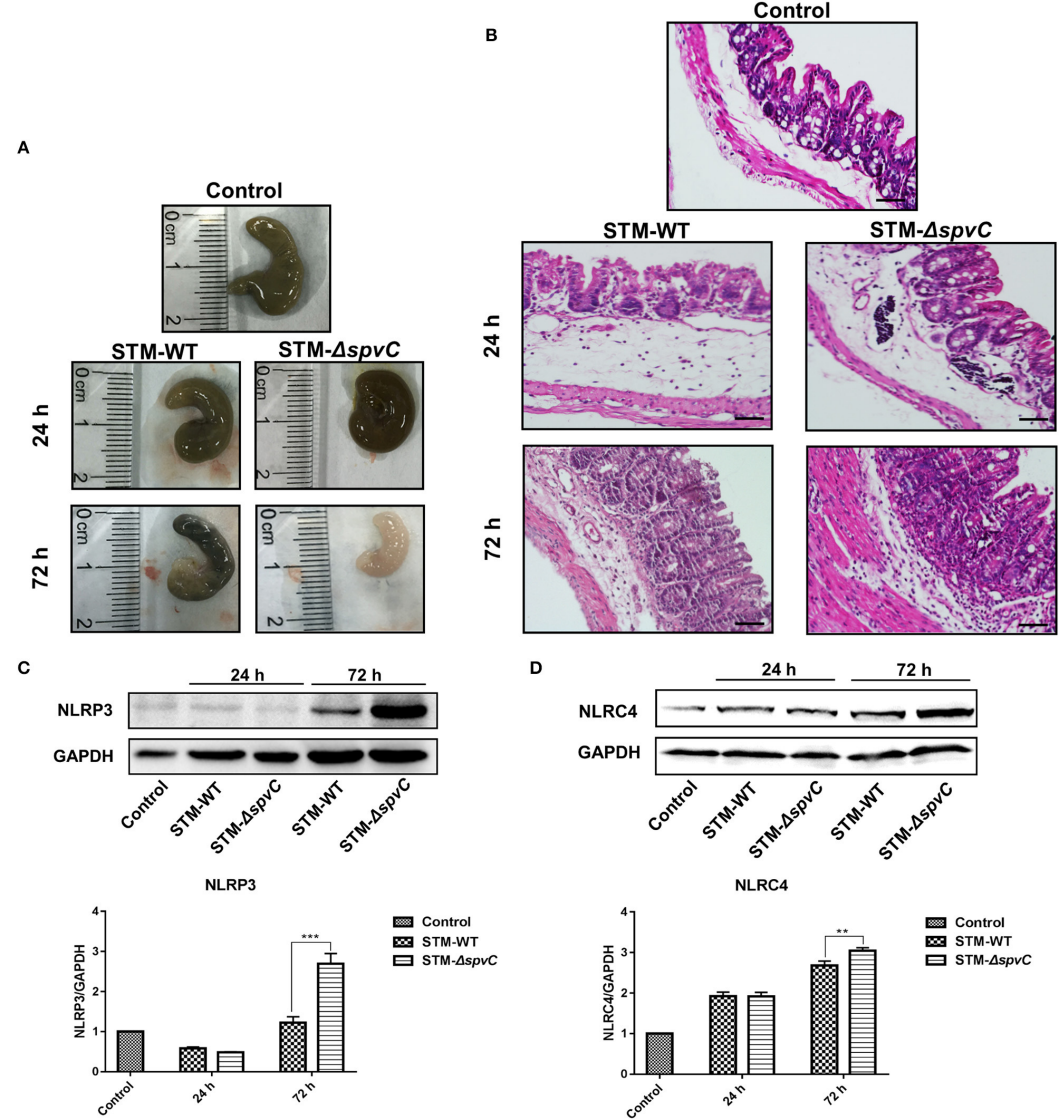
Effects of *spvC* on *Salmonella* pathogenesis in intestinal inflammatory response. Mice were infected orally with 1 × 10^8^ colony-forming unit (CFU) of either the STM-WT or the STM-Δ*spvC* and analyzed at 24 and 72 hpi. **(A)** Macroscopic pictures of the ceca. **(B)** Histopathological analysis of the ceca; scale bars, 50 μm. **(C)** Western blot analysis of whole cecum with specific antibodies to NLRP3 and the control GAPDH. **(D)** Western blot analysis of whole cecum with specific antibodies to NLRC4 and the control GAPDH. Data were compared by independent Student's *t*-test. Values are expressed as the means ± SD, and statistically significant differences are indicated. ***P < 0.001; **P < 0.01. Data were from at least three experiments.

A streptomycin-pretreated murine model was commonly used for *Salmonella* diarrhea (Kaiser et al., 2012). Thus, we developed a streptomycin-pretreated murine model to investigate *Salmonella*-induced intestinal inflammation. A previous study reported that *spvC* was required by *Salmonella* to modulate intestinal inflammatory response at 48 h post *S*. Typhimurium infection (Haneda et al., 2012). To further investigate the dynamic effect of *spvC* in intestine, we evaluated the pathological changes of the ceca in streptomycin-pretreated mice after *S*. Typhimurium infection. The expression of *spvC* in STM-c-*spvC* is induced by l-arabinose (0.2%); this condition could be fulfilled *in vitro*, while could not be ensured for *in vivo* assay. Thus, mice were infected orally with either the STM-WT or the STM-Δ*spvC* in our study. Macroscopic pictures of the ceca are shown in Figure 1A. The ceca length of mice infected with *S*. Typhimurium contracts slightly at 24 hpi compared with that of mice in the control group, while the marked contraction of the ceca is observed at 72 hpi. Importantly, shorter cecum is observed in STM-Δ*spvC*-infected mice than in STM-WT-infected mice at 72 hpi. Histopathological examination of the ceca in these mice supports the above results (Figure 1B). At 24 hpi, mice infected with *S*. Typhimurium show weak pathological changes in their ceca including slight swelling and infiltration of inflammatory cells into the lamina propria and submucosa compared with those in the uninfected group. The lesions aggravate at 72 hpi. Pronounced cecal inflammation is also observed in mice infected with STM-Δ*spvC* than in those infected with STM-WT, including epithelial cell swelling and inflammatory cell infiltration in the lamina propria, as well as atrophy in the submucosa. Together, these results indicate that the deletion of *spvC* leads to an exacerbation of the inflammatory response.

PAMPs can function as stimuli to activate inflammatory signaling receptor NLRP3 and NLRC4 (Karki et al., 2018; Swanson et al., 2019). Although the roles of NLRP3 and NLRC4 in intestinal immune defense against *S*. Typhimurium invasion have been studied, their precise functions of *spvC*-mediated *Salmonella* infection in the gut remain elusive (Li et al., 2017; Rauch et al., 2017). To further investigate the underlying mechanisms, NLRP3 and NLRC4 in the ceca of mice infected with *S*. Typhimurium strains were analyzed by western blot (Figures 1C,D). Results show the increased expression of NLRP3 and NLRC4 at 72 hpi compared with those in the control groups. Notably, higher protein levels of NLRP3 and NLRC4 are found in STM-Δ*spvC*-infected mice than those in STM-WT-infected mice. These data suggest that *spvC* regulates NLRP3 and NLRC4 negatively, which may relate to the inhibition of intestinal inflammation.

### *spvC* Mediates *Salmonella*-Suppressing Pyroptosis in Macrophages via Its Phosphothreonine Lyase Activity

**Figure 2:**
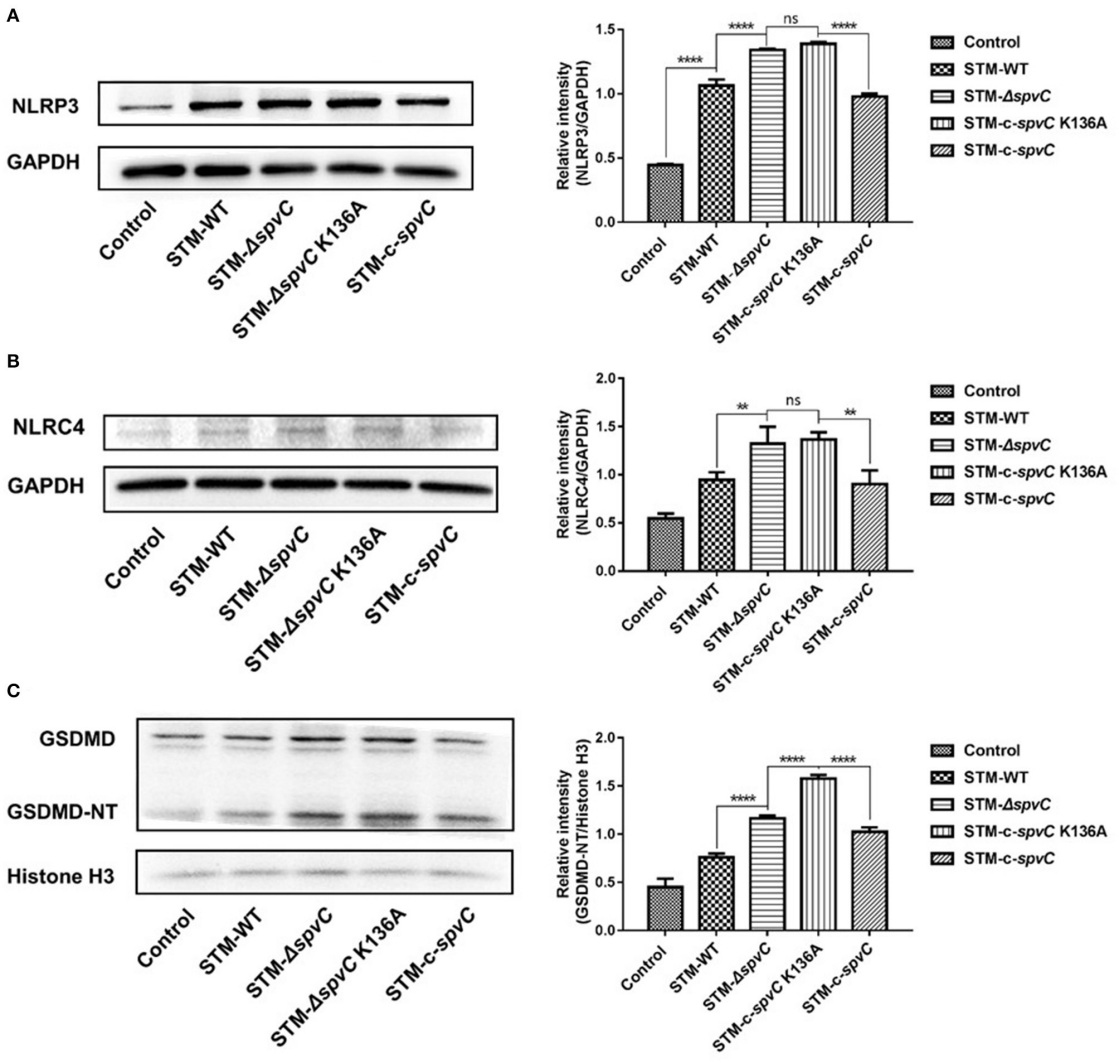
*spvC* mediates *Salmonella*-suppressing pyroptosis in macrophages via its phosphothreonine lyase activity. J774A.1 cells were infected with STM-WT, STM-Δ*spvC*, STM-c-*spvC* K136A, and STM-c-*spvC* and analyzed at 8 hpi. **(A–C)** Western blot analysis of cell lysates with specific antibodies to NLRP3, NLRC4, GSDMD, and the control GAPDH or histone H3. Data were compared by one-way ANOVA. Values are expressed as the means ± SD, and statistically significant differences are indicated. ****P < 0.0001; **P < 0.01; ns, not significant. Data were from at least three experiments.

Pyroptosis that could be activated by NLRP3 and NLRC4 strengthened host defense function by its restriction of intracellular bacteria through PITs and disruption of the pathogen replication niche (Shi et al., 2017; Evavold et al., 2018; Karki et al., 2018). Consequently, pathogens as well as PITs released to extracellular space were available to be eliminated by phagocytes and downstream cell intrinsic defenses (Kovacs and Miao, 2017). It is known that *spvC* encoding protein, SpvC, is a *Salmonella* effector with phosphothreonine lyase activity. To investigate whether the enzymatic activity of SpvC is involved in its effect on pyroptosis, J774A.1 cells were co-cultured with STM-WT, STM-Δ*spvC*, STM-c-*spvC* K136A, which is a directed mutant in the phosphothreonine lyase activity site and STM-c-*spvC* for 8 hpi. As expected, the levels of NLRP3 and NLRC4 were significantly increased after infection with *S*. Typhimurium. Both STM-Δ*spvC* and STM-c-*spvC* K136A give rise to elevated levels of NLRP3 and NLRC4 in their infected macrophages compared with *S*. Typhimurium-carrying *spvC*-infected cells. However, the same levels of NLRP3 and NLRC4 were detected between cells infected with STM-Δ*spvC* and STM-c-*spvC* K136A (Figures 2A,B). We next assessed GSDMD, an executor of pyroptosis and its pore-forming domain GSDMD-NT. In line with the changing trend of NLRP3 and NLRC4, levels of GSDMD-NT are significantly higher in cells infected with STM-Δ*spvC* and STM-c-*spvC* K136A than in those infected with STM-WT and STM-c-*spvC* (Figure 2C). Moreover, J774A.1 cells were co-cultured with STM-WT, STM-Δ*spvC*, and STM-c-*spvC* after being pretreated with MAPK inhibitors (ERK inhibitor PD98059, JNK inhibitor SP600125, and P38 inhibitor SB203580) to study the change of NLRP3 and NLRC4 protein levels (Supplementary Figure 1). The aforementioned results demonstrate that the phosphothreonine lyase activity of SpvC is required to inhibit pyroptosis.

### *spvC* Attenuates Pyroptosis in the Ceca in Host Defense Against *Salmonella* Typhimurium Infection

**Figure 3:**
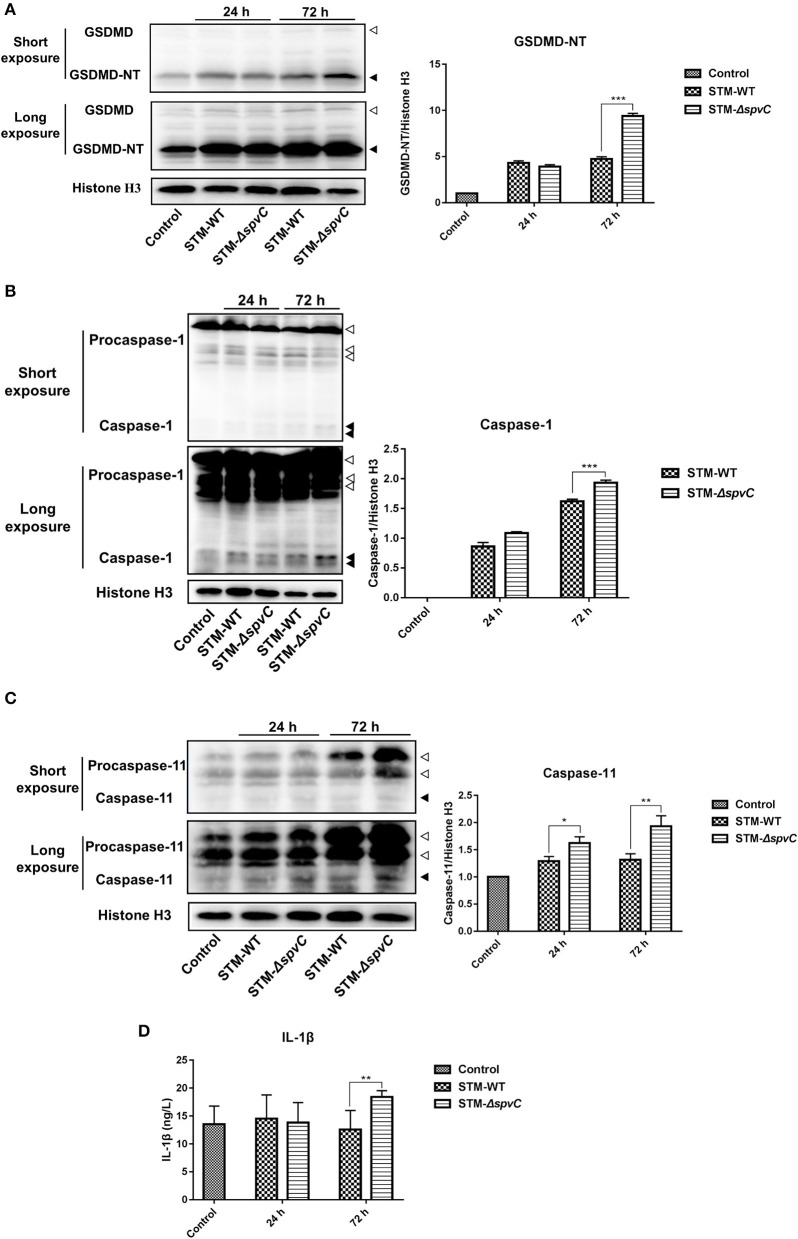
*spvC* attenuates pyroptosis in the ceca in host defense against *Salmonella* infection. Mice were infected orally with 1 × 10^8^ colony-forming unit (CFU) of either the STM-WT or the STM-Δ*spvC* and analyzed at 24 and 72 hpi. Mice uninfected with *Salmonella* served as control groups. **(A)** Western blot analysis of whole cecum with specific antibodies to GSDMD and the control histone H3. White arrowheads indicate GSDMD, and black arrowheads indicate GSDMD-NT. **(B)** Western blot analysis of whole cecum with specific antibodies to Caspase-1 and the control histone H3. White arrowheads indicate Procaspase-1, and black arrowheads indicate Caspase-1. **(C)** Western blot analysis of whole cecum with specific antibodies to Caspase-11 and the control histone H3. White arrowheads indicate Procaspase-11, and black arrowheads indicate Caspase-11. **(A–C)** Data were from at least three experiments. **(D)** ELISA analysis of IL-1β in peripheral blood serums. Control group, *n* = 2; STM-WT 24 hpi group, *n* = 6; STM-Δ*spvC* 24 hpi group, *n* = 5; STM-WT 72 hpi group, *n* = 4; STM-Δ*spvC* 72 hpi group, *n* = 6. Data were compared by independent Student's *t*-test. Values are expressed as the means ± SD, and statistically significant differences are indicated. ***P < 0.001; **P < 0.01; *P < 0.05.

To determine whether *spvC* attenuates pyroptosis in the ceca against *S*. Typhimurium invasion, western blot analysis reveals that the expression of GSDMD-NT in *S*. Typhimurium-infected mouse ceca is five-fold of that in the control group. Moreover, the deletion of *spvC* results in an increased expression of GSDMD-NT in the ceca (Figure 3A). These results indicate that *spvC* could attenuate pyroptosis in the ceca.

Two distinct pathways, named the canonical and non-canonical inflammasome pathways, initiate the assembly of Caspase-1 and Caspase-11 in mice, respectively, which in turn cleave GSDMD (Broz et al., 2020). To investigate which inflammasome pathway was involved in this model, we subsequently determined the hallmarks active fragments of Caspase-1 and Caspase-11. In line with the results obtained in Figure 3A, significantly more cleavage of Caspase-1 and Caspase-11 is determined in *S*. Typhimurium-infected ceca than that in the control groups. The expression of Caspase-1 and Caspase-11 in the ceca of mice infected with STM-Δ*spvC* is significantly higher than that in STM-WT-infected mice at 72 hpi (Figures 3B,C). These results reveal that *spvC* subverts cecal pyroptosis through both canonical and non-canonical inflammasome pathways.

In addition to cleaving GSDMD to render them bioactive and to induce pyroptotic cell death, auto-processed Caspase-1 matures the pro-inflammatory cytokine IL-1β and consequently triggers a robust inflammatory response (Gaidt and Hornung, 2016). Based on this, we find an increasing evidence of the IL-1β secretion in serum at 72 hpi due to the absence of *spvC*, which is consistent with the level of GSDMD-NT protein (Figure 3D). These findings suggest that *spvC* suppresses the secretion of IL-1β and attenuates host immune defense against infection.

### *spvC* Is Required for Inflammatory Injury and *Salmonella* Typhimurium Dissemination

**Figure 4:**
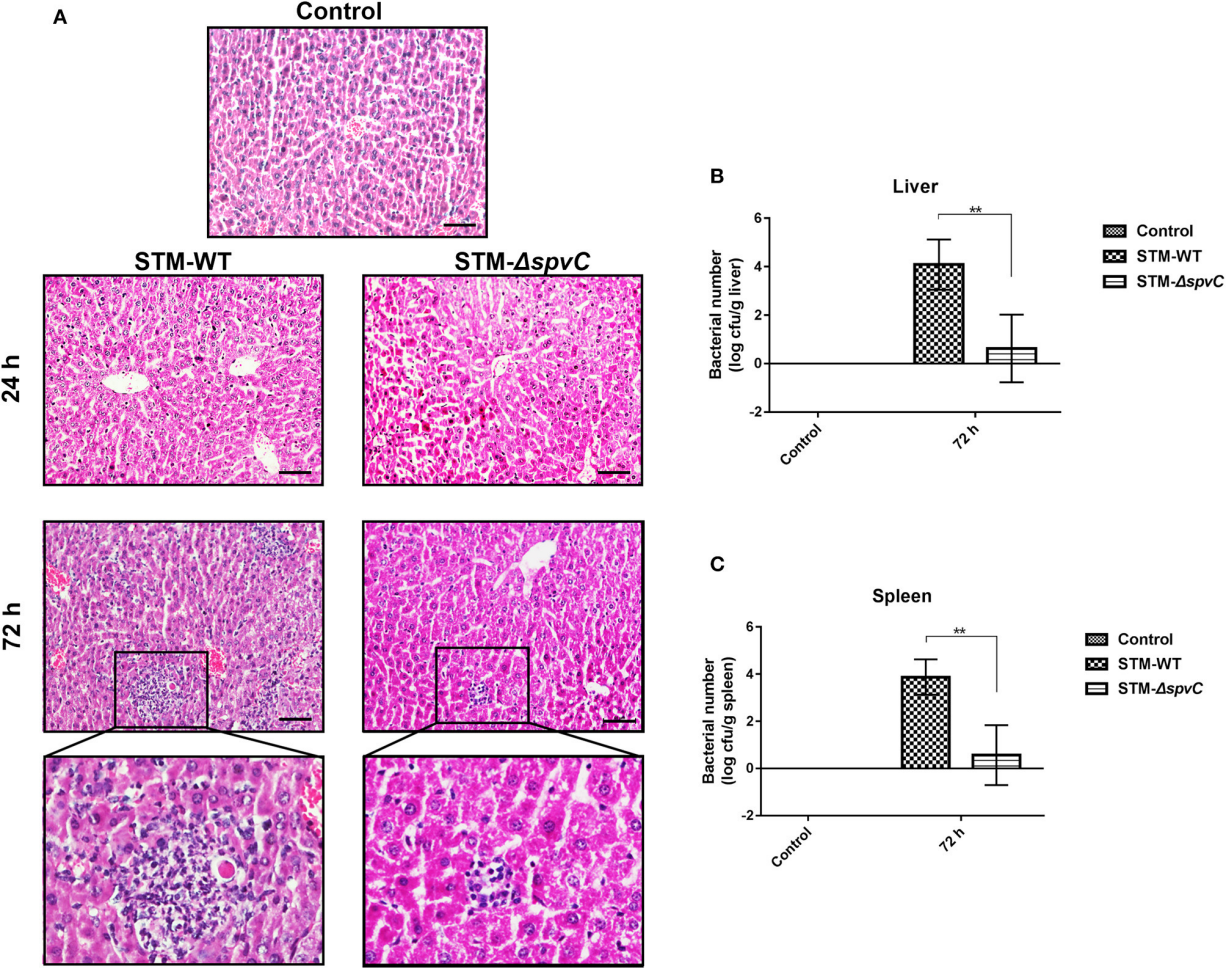
*spvC* is required for *Salmonella* dissemination and inflammatory injury. Mice were infected orally with 1 × 10^8^ colony-forming unit (CFU) of either the STM-WT or the STM-Δ*spvC* and analyzed at 24 and/or 72 hpi. **(A)** Histopathological analysis of liver; scale bars, 50 μm. Data were from at least three experiments. **(B)** The bacterial load of livers. **(C)** The bacterial load of spleens. **(B,C)** Control group, *n* = 2; STM-WT 72 hpi group, *n* = 4; STM-Δ*spvC* 72 hpi group, *n* = 5. Data were compared by independent Student's *t*-test. Values are expressed as the means ± SD, and statistically significant differences are indicated. **P < 0.01.

Reduced production of IL-1β and intestinal inflammatory response are responsible for *S*. Typhimurium systemic infection. A previous study demonstrated that a mutation in *spvC* did not affect bacterial colonization in the liver of mice infected with *S*. Typhimurium at 24 hpi, which was in agreement with our results (Haneda et al., 2012). However, with lastingness of infection, we find the exacerbation of inflammatory lesions in the liver of mice infected with *S*. Typhimurium as compared with control group. Moreover, mice infected with STM-WT have a more severe hepatic inflammation such as inflammatory cell infiltration in the hepatic lobules than those infected with STM-Δ*spvC* at 72 hpi (Figure 4A). These results indicate that *spvC* is required for hepatic injury during *S*. Typhimurium infection. Concomitantly, much more bacteria (~10^4^ CFU/g) from livers are found in mice infected with *S*. Typhimurium-carrying *spvC* than those infected with STM-Δ*spvC* (Figure 4B). We further examine the bacterial loads in the spleen and find that the bacterial number in the spleen coincides with that in the liver (Figure 4C). These data confirm that *spvC* is required for *S*. Typhimurium dissemination in systemic organs.

## Discussion

*Salmonella* are primary enteric pathogens infecting both humans and animals, among which *S*. Typhimurium is one of the most common isolates in clinical practice (Keestra-Gounder et al., 2015). After ingestion, *S*. Typhimurium invade preferentially in the cecum and terminal ileum in mice (Kaiser et al., 2012). A previous study showed that *spvC* could restrain intestinal inflammatory response during the early stages of infection in BALB/c mice infected with *Salmonella* strain 14,028 (Haneda et al., 2012). While the interaction between bacteria and host is very complex, *Salmonella*-induced intestinal inflammatory response may be affected by different virulent strains and host background genetics. In this study, we established the *in vivo* model using C57BL/6 mice infected with *Salmonella* strain SL1344. Data are in line with the earlier report. In a word, *spvC* exerts universal anti-inflammatory effect to pathogenesis of *S*. Typhimurium. To assess a possible correlation between intestinal inflammation and the onset of systemic infection, we extend these studies by monitoring the time course of length and pathology of the ceca. As expected, the inflammatory lesions in the lamina propria and submucosa of the ceca in mice exacerbated at 72 hpi. This suggests that *spvC* time dependently modulates host immune response by downregulating intestinal inflammation.

Inflammasomes are signaling hubs that activate inflammatory signaling cascades to drive host defense against invading pathogens. A previous study revealed that SPI2 T3SS could disrupt NLRP3 and NLRC4 inflammasome responses (Bierschenk et al., 2017). We further demonstrate that *spvC*, the effector of SPI2 T3SS, downregulates the expression of NLRP3 and NLRC4 in the ceca of mice infected with *S*. Typhimurium. Moreover, NLRP3 is uniquely activated by a wide variety of stimuli, including microbial motifs, endogenous danger signals, and environmental irritants (Swanson et al., 2019). In contrast, NLRC4 is mostly triggered by cytosolic flagellin (Duncan and Canna, 2018). This discrepancy probably leads to the susceptibility of NLRP3 in response to *Salmonella* infection.

After entering the lamina propria at the site of Peyer's patches, *S*. Typhimurium are taken up by phagocytes (Broz et al., 2012). Migration of these infected phagocytes, predominantly macrophages, facilitates systemic dissemination of the bacteria via the bloodstream to several additional tissues, such as the spleen and liver. SpvC is a phosphothreonine lyase that exerts anti-inflammatory effects by inactivating dual-phosphorylated MAPK through beta elimination (Li et al., 2007). We identify the enzymatic activity of SpvC, contributing to suppress NLRP3, NLRC4, and GSDMD-NT in macrophages, which might confer an advantage to the pathogen during host–pathogen competition. Furthermore, MAPK can transmit signals from the cell membrane to the nucleus, which may provide the first signal for transcription of inflammasomes (Zhao et al., 2019). So MAPK signaling pathway may be involved in the inhibitory effect of *spvC* on NLRP3 and NLRC4, subsequently suppressing pyroptosis.

Our previous study revealed that *S*. Typhimurium could cause PCD in several different ways including apoptosis and autophagy for pathogen clearance (Wu et al., 2010; Chu et al., 2014). Besides, *Salmonella* outer protein B (SopB) suppresses colitis development and increases bacteria pathogenesis via modulating necroptosis (Hu et al., 2019). Notably, experimental evidence reveals that *S*. Typhimurium trigger pyroptosis through both Caspase-1- and Caspase-11-dependent manner (Shi et al., 2015). In order to investigate the effect of *spvC* on pyroptosis in the ceca, GSDMD-NT, Caspase-1, and Caspase-11 were detected. The expression of GSDMD-NT, Caspase-1, and Caspase-11 in the ceca of mice infected with STM-Δ*spvC* increases to a comparable level to those in STM-WT-infected mice. This suggests that *spvC* inhibits pyroptosis through both canonical and non-canonical inflammasome pathways. Pyroptosis elicits a potent innate immune response resulting in IL-1β secretion (He et al., 2015). This effect functions as a robust host defense by recruiting secondary immune cells working as “scavengers” engulfing either infected dying cells or bacteria released into the extracellular space (Jorgensen et al., 2016a). Of interest, we find that the IL-1β level in serum of mice infected with *S*. Typhimurium without *spvC* is significantly higher than that in *S*. Typhimurium-carrying *spvC*-infected mice. These findings suggest that *spvC* decreases IL-1β secretion, which may attenuate host defense. This mechanism was probably the primary cause of the systemic infection by *S*. Typhimurium. Intriguingly, Caspase-8, a molecular switch for apoptosis, necroptosis, and pyroptosis, regulates an additional pathway controlling GSDMD-driven pyroptosis in *Yersinia*-infected macrophages (Orning et al., 2018). Whether the Caspase-8/GSDMD pathway is also involved in *Salmonella* infection deserves deep investigation.

Although gastroenteritis is a localized infection of the intestinal mucosa and mesenteric lymph nodes, the enhanced production of pro-inflammatory cytokines induces acute inflammation and is required for *Salmonella* clearance (Pradhan et al., 2020). We speculated that the effect of *spvC* on suppressing IL-1β secretion and attenuating host defense may lead to *S*. Typhimurium dissemination. Herein, we find that the lesions in livers of mice infected with STM-Δ*spvC* are slighter than those in STM-WT-infected mice. The viable bacteria isolated from murine livers and spleens infected with STM-WT at 72 hpi are about than 10^4^ of those in STM-Δ*spvC*-infected mice. Hence, the spread of *S*. Typhimurium to the liver and spleen, where host defense limits bacterial replication and eliminates pathogen, is eventually increased by *spvC*. From the host's perspective, NAIP/NLRC4 inflammasome in intestinal epithelial cells (IECs) can prevent systemic dissemination of *S*. Typhimurium *in vivo* (Hausmann et al., 2020). To escape from the host, *Salmonella* in part overcomes this negative regulation of reverse transmigration into the bloodstream with *spvC* (Gopinath et al., 2019).

## Conclusion

In summary, *Salmonella* infection models *in vivo* and *in vitro* were used to investigate potential pathogenesis of *spvC* gene via modulation of pyroptosis and intestinal inflammation, thereby increasing systemic dissemination. The present study demonstrates that SpvC alleviates intestinal inflammation, downregulates the expression of proteins related to pyroptosis through its enzymatic activity, subsequently inhibits cecal cell pyroptosis through both canonical and non-canonical inflammasome pathways, regulates the secretion of pro-inflammatory cytokines, and finally promotes bacterial dissemination in mice. These findings reveal a novel contribution of *spvC* to pathogenesis of *S*. Typhimurium by inhibiting pyroptosis and intestinal inflammation, and this effect may associate with immune strategy of systemic dissemination. Our research has potentially important significance to provide new paradigms for interactions between bacteria and host immune response, which would provide novel insights for controlling *Salmonella* infection and other infectious diseases.

## Data Availability Statement

The original contributions presented in the study are included in the article/Supplementary Materials, further inquiries can be directed to the corresponding authors.

## Ethics Statement

The animal study was reviewed and approved by the Animal Experimental Committee of the Soochow University.

## Author Contributions

LZu, LZh, and SW designed the research and wrote the manuscript. LZu, LZh, CW, and YW performed the research and conducted the data analysis. YL, RH, and SW supervised the project and edited the manuscript. All authors have read and approved the manuscript.

## Conflict of Interest

The authors declare that the research was conducted in the absence of any commercial or financial relationships that could be construed as a potential conflict of interest.
