# Supplementary material for: Salmonella spvC Gene Inhibits Pyroptosis and Intestinal Inflammation to Aggravate Systemic Infection in Mice
Source: Front Microbiol. 2020 Dec 15;11:562491. doi: 10.3389/fmicb.2020.562491 (PMC7770238; doi:10.3389/fmicb.2020.562491)
Supplement: Supplementary file 1 [file Data_Sheet_1.docx]

**Supplementary materials**

| **TABLE S1: Primers used for construction and identification of strains** | |
| --- | --- |
| Name | Nucleotide sequence (5' to 3')*^a^* |
| H_1_P_1_ | TTTATATCTAATAATATGGCGATATATCCATATCGCAAAGGAGATTTCCCGTGTAGGCTGGAGCTGCTTC |
| H_2_P_2_ | AAATAGCTGTTTAACGGCGTTTACTGTTCCGTTGCTCCCCAAACCCATACATGGGAATTAGCCATGGTCC |
| F(*Xho* I) | CCTCGAGCCCATAAATAGGCCTAATCT |
| R(*Eco*R I) | GGAATTCCCTCTGTCATCAAACGATAAA |
| P3 | TGAGGTTTTCTGGATAACTAATCG |
| P4 | TGCGGACATATCAATATGCATGAG |
| F (*spvC* K136A) | GACAGTCCGGTAGATAAGTGGGCAGTGACCGATATGGAGAAGGTCGTTCAACAAGCCCGT |
| R (*spvC* K136A) | CGACCTTCTCCATATCGGTCACTGCCCACTTATCTACCGGACTGTCCTCTGAAAACAGCA |
| *^a^* Underling indicates homologous arms. Sequences in the boxes are restriction sites. | |


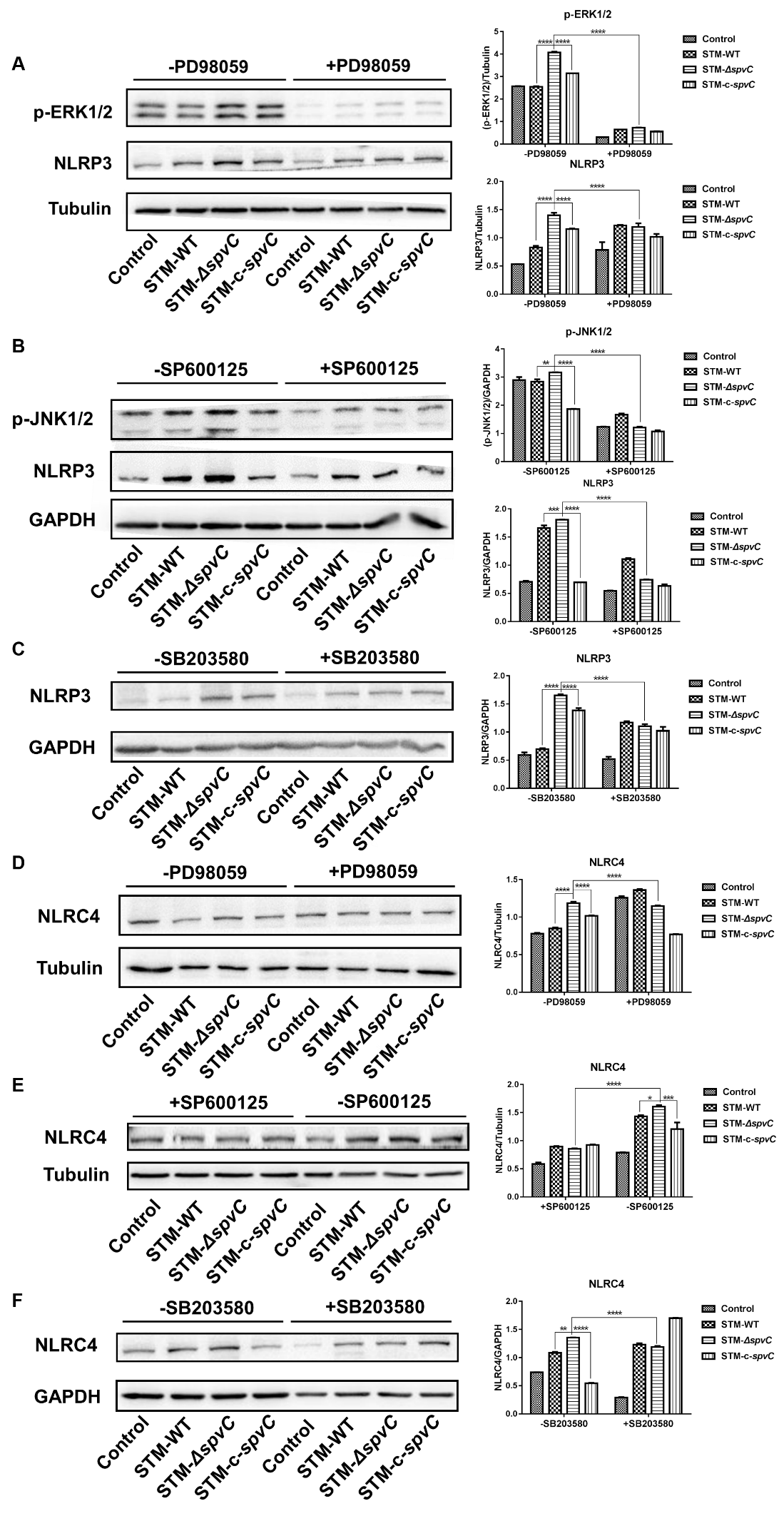


**FIGURE S1** *spvC* mediates *Salmonella* suppressing pyroptosis in macrophages via its phosphothreonine lyase activity. J774A.1 cells were infected with STM-WT, STM-*ΔspvC* and STM-c-*spvC* after pre-treated with 10 µM PD98059 (ERK inhibitor), SP600125 (JNK inhibitor), SB203580 (P38 inhibitor) for 3 h and analyzed at 8 hpi. **(A-F)** Western blot analysis of cell lysates with specific antibodies to phospho-ERK1/2, phospho-JNK1/2, NLRP3, NLRC4 and the control Tubulin or GAPDH after pre-treated with respective inhibitors. Data were compared by one-way ANOVA within groups and two-way ANOVA between groups. Values are expressed as the means ± S.D. and statistically significant differences are indicated. *****P* < 0.0001; ****P* < 0.001; ***P* < 0.01; **P* < 0.05. Data were from at least three experiments.
